# Caveolin-1 knockout mitigates breast cancer metastasis to the lungs via integrin α3 dysregulation in 4T1-induced syngeneic breast cancer model

**DOI:** 10.1038/s41417-024-00821-4

**Published:** 2024-09-07

**Authors:** Dhirendra Pratap Singh, Rashmi Pathak, Naveen Chintalaramulu, Abhishek Pandit, Avinash Kumar, Philip J. Ebenezer, Sanjay Kumar, Alexander Duplooy, Mary Evelyn White, Nithya Jambunathan, Rohan Dharmakumar, Joseph Francis

**Affiliations:** 1 Krannert Cardiovascular Research Center, Indiana University School of Medicine, Indianapolis, IN USA; 2 School of Veterinary Medicine, Louisiana State University, Baton Rouge, LA USA; 3 Department of Biological Sciences Louisiana State University, Baton Rouge, LA USA; 4 College of Veterinary Medicine, Midwestern University, Glendale, AZ USA

**Keywords:** Cell biology, Cancer, Breast cancer

## Abstract

Caveolin-1 (Cav-1) is a critical lipid raft protein playing dual roles as both a tumor suppressor and promoter. While its role in tumorigenesis, progression, and metastasis has been recognized, the explicit contribution of Cav-1 to the onset of lung metastasis from primary breast malignancies remains unclear. Here, we present the first evidence that Cav-1 knockout in mammary epithelial cells significantly reduces lung metastasis in syngeneic breast cancer mouse models. In vitro, Cav-1 knockout in 4T1 cells suppressed extracellular vesicle secretion, cellular motility, and MMP secretion compared to controls. Complementing this, in vivo analyses demonstrated a marked reduction in lung metastatic foci in mice injected with Cav-1 knockout 4T1 cells as compared to wild-type cells, which was further corroborated by mRNA profiling of the primary tumor. We identified 21 epithelial cell migration genes exhibiting varied expression in tumors derived from Cav-1 knockout and wild-type 4T1 cells. Correlation analysis and immunoblotting further revealed that Cav-1 might regulate metastasis via integrin α3 (ITGα3). In silico protein docking predicted an interaction between Cav-1 and ITGα3, which was confirmed by co-immunoprecipitation. Furthermore, Cav-1 and ITGα3 knockdown corroborated its role in metastasis in the cell migration assay.

## Introduction

Breast cancer (BC) remains the most prevalent malignancy among women worldwide, and as of 2022, it is the second leading cause of cancer-related death [1]. The majority of fatalities in BC are caused by metastasis, which accounts for nearly 90% of deaths associated with the disease [2–7]. Despite early detection and treatment advances, metastatic BC remains a significant clinical challenge.

The metastatic spread of BC is enabled by complex interactions between tumor cells and the microenvironment of distant organs [8]. A key step is the formation of a premetastatic niche in target organs, which is facilitated by tumor-secreted factors and signaling pathways [9]. The complexity of these interactions is highlighted by recent studies revealing the role of Toll-like receptor 4 (TLR4) in creating premetastatic niches for metastatic cancer stem cells [10]. Critical regulators of metastasis via tumor microenvironmental effects include the CXCR7 signaling axis, which promotes the growth and progression of BC [11]. Furthermore, the importance of the tumor microenvironment and metastatic niche in cancer progression is underscored by their ability to suppress metastases [9, 12, 13]. These findings indicate that the metastatic journey of BC cells is shaped by crosstalk between the tumor and its microenvironment at distant sites, which requires the formation of a premetastatic niche.

Caveolin-1 (Cav-1), a protein associated with lipid rafts, plays various roles in cellular processes such as signal transduction and angiogenesis. Its role in cancer is complex, acting both as a tumor suppressor and as an oncogene, depending on the type and stage of cancer [14–20]. Aberrant Cav-1 expression in BC suggests its involvement in disease progression [14, 21–23]. This is particularly evident in triple-negative breast cancer (TNBC), a subtype characterized by the absence of estrogen, progesterone, and HER2 receptors, and is associated with a poor prognosis and high recurrence rates [24, 25]. Studies have shown that downregulation of Cav-1 in TNBC cells hampers the formation of invadopodia and lung colonization upon intravenous injection into the tail vein of BALB/c mice, highlighting its potential role in metastasis [25, 26]. Furthermore, Cav-1 polymorphisms have been associated with an increased risk of recurrence and contralateral BC [27].

Despite considerable research, the specific contribution of Cav-1 to BC metastasis is not fully understood. Sloan et al. highlighted the prognostic importance of stromal Cav-1 expression, noting that its loss in tumors is strongly related to increased metastasis and poorer outcomes [28]. Furthermore, Burrows et al. suggested that Cav-1 expression in mammary fibroblasts could suppress BC cell growth and migration, indicating its potential tumor suppressor role [29]. Their research revealed a regulatory effect of Cav-1 on cytokine secretion, such as IL-6, from stromal cells, which could influence the metastatic processes. Wang et al. explored how tumor-derived Cav-1 contributes to lung metastasis and discovered that it can be transported via exosomes to metastatic sites, where it modulates gene expression related to premetastatic niche formation and secretion of inflammatory chemokines in lung epithelial cells. Additionally, Cav-1 promotes the deposition of the extracellular matrix by lung fibroblasts and facilitates angiogenesis by inducing M2 macrophage polarization [25].

Integrin-α3 (ITGα3), a subunit of integrin receptors and key facilitator of cell adhesion and migration, is highly expressed in malignant cancers. It is important to note that Cav-1 and ITGα3 play significant roles in various cellular processes, including cell adhesion, migration, and signaling pathways, which are crucial in both normal physiology and disease states. Silencing ITGα3 expression in pancreatic cancer cells significantly inhibits cell viability and migration, indicating its role in cancer aggressiveness [30]. Similarly, in intrahepatic cholangiocarcinoma (ICC), ITGα3 overexpression is associated with adverse clinicopathological features and poor survival outcomes, further underscoring its oncogenic potential [31]. Notably, in hepatocellular carcinoma, Cav-1 enhances invasion and metastasis by upregulating Pofut1 expression [32], suggesting a broader oncogenic role. Collectively, these insights underscore Cav-1’s multifaceted involvement in the promotion of metastasis in different types of cancers. However, the reliance on in vitro systems and intravenous injection models in these studies, although informative, may not fully capture intricate biological interactions occurring in vivo [33, 34]. These findings indicate that Cav-1 and ITGα3 are integral to the regulation of cellular processes and might contribute to lung metastasis in the BC model.

In this study, we aimed to elucidate how Cav-1 influences the molecular mechanisms underlying TNBC metastasis. We employed the CRISPR/Cas9 gene editing tool to generate Cav-1 deficient 4T1 murine mammary carcinoma cells. By implanting Cav-1 KO and WT 4T1 cells into female BALB/c mice, we examined the effect of Cav-1 ablation on TNBC progression and lung metastasis in primary tumors. In-depth gene expression analysis after Cav-1 knockout provided further insights into the molecular underpinnings of its role in facilitating metastasis. A significant component of our study was the examination of the interaction between Cav-1 and ITGα3. Our principal goal was to decipher the complex mechanisms by which Cav-1 promotes the dissemination of BC cells, emphasizing its effects on the premetastatic signaling pathways.

## Materials and methods

### Cell culture and development of Cav-1 genetic knockout cells

4T1 carcinoma cells, derived from the mammary gland tissues of BALB/c mice (ATCC, Cat No# CRL-2539™), were cultured in RPMI-1640 medium (ATCC, Cat#30-2001™) supplemented with 10% Fetal Bovine Serum (FBS, Thermofisher Cat# 10438026) and 1% v/v Penicillin-Streptomycin (Cat#2020-06-30). Cells were maintained at 37 °C in a 5% CO_2_ incubator. Cav-1 gene deletion was performed using a pre-designed and validated Mouse CAV1 Gene Knockout Kit (OriGene Technologies, Cat#: KN502589), according to the manufacturer’s instructions. In brief, 0.3 × 10^6^ cells were seeded in a 6-well plate and incubated for 24 h in a CO_2_ incubator. Upon reaching 70–80% confluency, cells were transfected with a plasmid harboring gRNA targeting Cav-1 (KN502589G1 or KN502589G2) and a GFP-tagged donor vector using TurboFectin 8.0 Transfection Reagent (CAT#: TF81001). Following a 48-h incubation, the cells were diluted in a 1:10 ratio and grown for 3 days. Cells were seeded at an extremely low density in 6-well plates to obtain single-cell colonies and were allowed to grow for 5 days. Subsequently, individual cells were selected under a microscope using a 20 μL pipette tip and cultivated into colonies. Each resulting colony was split into two groups in the following week: one for further expansion and the other for verification of Cav-1 KO by immunoblotting. Multiple single-cell Cav-1 KO and WT cells were isolated after the same number of passages. Purified Cav-1 KO and WT 4T1 cells were used for subsequent in vitro and in vivo experiments.

### MTT assay

To assess the impact of Cav-1 KO on cell growth, we performed an MTT assay using the Vybrant® MTT Cell Proliferation Assay Kit (V-13154) according to the manufacturer’s instructions. Briefly, 5000 WT and Cav-1 KO 4T1 cells were seeded in a 96-well plate and allowed to grow for 48 h. After this period, 10 µL of MTT solution (3-(4,5-dimethylthiazol-2-yl)-2,5-diphenyltetrazolium bromide) from a 12 mM stock was added to each well containing 100 µL of cell culture medium, followed by incubation at 37 °C for 2 h. Subsequently, 100 µL of 100 mg/mL SDS solution in 0.01 M HCl was added to each well, and the plate was incubated at 37 °C for an additional 2 h. Absorbance was measured at 570 nm, and a standard curve (absorbance vs. cell number) was generated to evaluate the effect of Cav-1 KO on cell growth in KO cells relative to the WT control.

### Immunoblotting

The peripheral zone of the tumor tissue was lysed in 1X RIPA buffer containing 1x protease and phosphatase inhibitor cocktail (ab201119, Abcam). The insoluble debris was removed by centrifugation at 16,000×*g* for 10 min at 4 °C. Protein quantification was performed using a Pierce™ BCA Protein Assay Kit (Thermo Scientific™, Cat#23225) according to the manufacturer’s instructions. Equal amounts of protein were mixed to make a final 1X Laemmli Sample Buffer (Cat# 161-0747) (Bio-Rad, Hercules, CA, USA) containing 2-mercaptoethanol and heated for 7–10 min at 95 °C. Approximately 15 mg of protein was loaded into each well and separated using SDS-PAGE. Proteins were electrotransferred onto a PVDF membrane (Bio-Rad, Hercules, CA, USA) and blocked with 5% skimmed milk for 45–90 min. After blocking, membranes were incubated overnight with primary antibodies against Cav-1 (Cat # sc-3238S, Santa Cruz Biotechnology), ITGα3 (Cat # sc-374242, Santa Cruz Biotechnology), and ITGβ4 (Cat # sc-514426, Santa Cruz Biotechnology). GAPDH from CST (Cat# 5174, Cell Signaling Technology) served as the loading control for the assay. HRP-labeled secondary antibody was used at a dilution of 1:2000. Blots were developed using ProSignal® Dura ECL Reagent (Cat# 20-301B, Prometheus Protein Biology Products) and visualized using a ChemiDoc Imaging System (Bio-Rad, Hercules, CA, USA). Band intensity was quantified using Image Lab software (Bio-Rad, Hercules, CA, USA).

### Mitochondrial stress assay

Approximately 2.5 × 10^4^ 4T1 cells were seeded in RPMI-1640 medium (Cat # 30-2001TM) supplemented with 10% FBS (Cat# 10438026) and 1% v/v penicillin-streptomycin (Cat # 2020-06-30) in Agilent Seahorse XF24 cell culture microplates (Cat# 100777-004), excluding four background temperature correction wells. The cells were incubated for 12–18 h at 37 °C in a 5% CO_2_ incubator. Sensor cartridges were hydrated with 200 μL of Seahorse XF calibrant and placed in a 37 °C humidified incubator without CO_2_ for 12–18 h. Fresh Seahorse assay medium was prepared and prewarmed at 37 °C. After incubation, the cells were gently washed three times with assay medium and then incubated in fresh assay medium for 1 h in a non-CO_2_, humidified 37 °C incubator. Oligomycin, FCCP (carbonyl cyanide 4-(trifluoromethoxy) phenylhydrazone), and rotenone/antimycin were diluted in assay medium to final working concentrations of 2.0 μM (oligomycin), 1.0 μM (FCCP), and 0.5 μM (rotenone/antimycin), respectively. Assay protocols were established as follows: baseline, 3 cycles; injection port A (oligomycin), 3 cycles; injection port B (FCCP), 3 cycles; and injection port C (Rotenone/antimycin A), 3 cycles. Each cycle consisted of an initial mix for 3 min, followed by a measurement of 3 min. After the cycles, data were acquired and normalized to the protein concentration. Wave software was used for data analysis, and normalization was performed based on protein concentration.

### Cell cycle assay

Approximately 0.5 × 10^6^ 4T1 cells were seeded in Corning™ Costar™ 6-well Clear TC-treated plates (Cat# 07-200-83) using RPMI-1640 (Cat#30-2001) complete medium and incubated overnight at 37 °C in a 5% CO_2_ incubator. After incubation, the cells were synchronized by replacing the complete medium with RPMI without FBS and incubating for 24 h. After the synchronization period, the FBS-free RPMI medium was replaced with a complete medium, and the cells were incubated for an additional 24 h. The cell cycle assay was performed using propidium iodide (PI) flow cytometry kit (ab139418) according to the manufacturer’s instructions. The cells were rinsed with 1X PBS, trypsinized, and collected in a complete medium. Cells were fixed in 66% ice-cold ethanol for 2 h at 4 °C and stained with 1X PI. PI fluorescence data were collected using flow cytometry with 488 nm laser illumination, utilizing appropriate forward and side scatter (FSC vs. SSC) settings.

### Scanning electron microscopy (SEM)

Polymethyl pentene plastic coverslips were used for cell growth in preparation for SEM analysis. Sterilized plastic coverslips were placed in a 6-well plate, and 0.5 × 10^6^ WT and Cav-1 KO 4T1 cells were seeded in complete media, followed by incubation at 37 °C in a 5% CO_2_ incubator. After 24 h, once the cells reached full confluence, they were washed with 1X PBS and fixed in a solution containing 1.25% glutaraldehyde and 2% formaldehyde in 0.1 M sodium cacodylate (CAC) buffer for 2 h at room temperature. The fixation buffer was replaced with a fresh fixation buffer and incubated at 4 °C. Subsequently, cells were washed with wash buffer (0.1 M CAC buffer with 5% sucrose), and post-fixation was performed using 1% osmium tetroxide in 0.1 M CAC buffer for 1 h. After fixation, cells were dehydrated using a series of ethanol concentration gradients. Following dehydration, the specimens were dried, mounted on a stub for metal coating, and subsequently analyzed using a JSM-6610 LV scanning electron microscope for membrane characterization.

### Transmission electron microscopy (TEM)

WT and Cav-1 KO 4T1 cells were grown and fixed using the same protocol as described in the SEM section. After fixation, the cells were washed and stained en bloc with 2% uranyl acetate in water at room temperature for 1 h. After en bloc staining, cells were dehydrated using a series of concentrations of ethanol (EtOH). Subsequently, the cells were infiltrated with epoxy resin and incubated at 60 °C for polymerization, which took 24–48 h. After polymerization, ultrathin sections of the specimens were obtained using a microtome. These sections were then observed under a JEM-1400 TEM optimized for high-contrast imaging at various magnifications.

### Exosome quantification

To quantify exosome secretion, equal numbers of 4T1, WT, and Cav-1 KO cells were plated in 6-well plates and allowed to grow for 24 h. After this initial growth period, the cells were cultured in a fresh medium devoid of fetal bovine serum (FBS) to eliminate potential exosome contamination from the serum. After an additional 24 h, exosomes were isolated from 1 mL of the conditioned media, and immunoblotting for exosome markers, Annexin-V, and Alix was performed.

### Tumor development in a syngeneic mouse model

Six-week-old female BALB/c mice were obtained from Jackson Laboratory and housed in the Division of Laboratory Animal Medicine of the LSU School of Veterinary Medicine for 4 weeks. At 10 weeks of age, the animals were weighed and randomly assigned to the WT group (mice that received WT-4T1 cells) or the Cav-1 KO group (mice that received Cav-1 KO-4T1 cells). Mice were anesthetized, and equal numbers of 4T1 cells (either WT or Cav-1 KO) were resuspended in Corning® Matrigel® basement membrane matrix (Corning, Cat#356237) and injected into the mammary fat pads of the mice. Tumor growth was monitored for four weeks after injection. Tumor and lung tissues were harvested at the end of weeks 2, 3, and 4. In a temporally controlled manner, peripheral tumor regions were excised at designated time points (weeks 2 and 3) to exclude centrally located necrotic tissue. The excised tissues were then subjected to mRNA expression profiling.

The lungs were perfused with 1% low-melting agarose and fixed with paraformaldehyde (PFA). Hematoxylin and eosin (H&E) staining was performed to examine lung metastasis in BC. Furthermore, to quantify the lung metastasis, single cell suspension was prepared using the fresh lungs from WT and Cav-1 KO, 4T1 injected mice after 3 weeks. These single-cell suspensions were allowed to grow in complete media containing 6-thioguanine (6-TG). After a week, the total number of growing cells was counted.

### RNA isolation, characterization, and sequencing

Total mRNA was isolated from the peripheral zone of the tumor using the Direct-zol RNA Kit (Cat# R2072) (Zymo Research, Irwin, CA) according to the manufacturer’s protocol. The RNA concentration was measured using a NanoDrop 1000 Spectrophotometer (Thermo Fisher Scientific Inc., Wilmington, DE, USA) at a wavelength of 260/280 nm. RNA quality and integrity were confirmed by agarose gel electrophoresis and fragment analyzer (Agilent 2100 Bioanalyzer). Samples with RNA Integrity Numbers (RIN) ranging from 6.7 to 9.3 were used for the preparation of the cDNA library and the sequencing of mRNA. Total RNA quality was verified at the Novogene Next-Generation Sequencing Facility. RNA samples with RIN values greater than six were selected, and mRNA enrichment was performed using poly T oligo-attached magnetic beads. After enrichment and fragmentation, cDNA libraries were prepared using the NEBNext Ultra II RNA Library Prep Kit for Illumina, following the manufacturer’s protocol. The cDNA library was quantified using Qubit and real-time PCR, and a bioanalyzer was used for size distribution analysis. Index-coded samples were clustered according to the manufacturer’s instructions, the library was sequenced on an Illumina platform (Illumina NovaSeq PE150), and paired-end reads were generated. Raw sequencing data could be found on NCBI SRA accession number PRJNA1098697.

### Sequencing data analysis

Illumina sequencing reads (fastq files) were initially processed using Perl scripts to remove reads containing adapters, poly-N sequences, and low-quality reads from raw data. Q20, Q30, and the GC content were also calculated. All downstream analyses were conducted using clean high-quality sequencing data. The reference genome and gene annotation models were obtained from the genome website, and the genome index was built using Hisat2 v2.0.5. Clean reads at the paired end were aligned with the reference genome using Hisat2 v2.0.5. Gene expression was quantified using featureCounts v1.5.0-p3, which counts the number of reads assigned to each gene. The FPKM values for each gene were calculated on the basis of the gene length and read counts. Sequenced library read counts were adjusted using the edgeR program package (version 3.22.5) with a single-scale normalized factor. Differential expression analysis between Cav-1 KO and WT tumors was performed using the DESeq2 R package (1.20.0). DESeq2 provided statistical routines for determining differential expression in digital gene expression data, and the obtained *P*-values were adjusted using Benjamini and Hochberg’s approach for controlling the false discovery rate. Gene length bias was corrected during Gene Ontology (GO) enrichment analysis using the clusterProfiler R package. GO terms with corrected *P*-values less than 0.05 were considered significantly enriched by differentially expressed genes. The Kyoto Encyclopedia of Genes and Genomes (KEGG) database was used to identify high-level functions of differentially expressed genes (DEGs). The statistical enrichment of DEGs in KEGG pathways was assessed using the clusterProfiler R package.

### Human BC data correlation analysis

The correlation between significant DEGs and Cav-1 was analyzed using the BC Gene-Expression Miner v4.8 (bc-GenExMiner), a disease-associated web portal that includes a BC DNA microarray, RNA sequencing, and clinicopathological databases. In the present study, targeted gene correlation analyses were performed on TNBC (IHC)/basal-like (PAM50) samples, focusing on the relationship between Cav-1 and 21 genes (Itgα3, Foxc2, Fermt1, Efnb2, Ptprm, Rhob, Card10, Ccbe1, Sox9, Cdh13, Plxnd1, Met, Flt4, Epha2, Rhoa, Kdr, Emp2, Ephb4, Mmrn2, Hdac5, and Epb41l4b). The gene expression correlations between Cav-1 and these 21 genes were analyzed in a cohort of 4,421 TNBC patients, and correlation graphs were generated using the same database.

### Prediction of in silico interaction between Cav-1 and ITGα3

The proximity of Cav-1 and ITGα3 to the membrane prompted us to investigate any potential interactions between these two proteins. The structural coordinates for Cav-1 and ITGα3 in Mus musculus were obtained using the AlphaFold deep-learning-based protein structure prediction program (https://alphafold.ebi.ac.uk/) [35]. AlphaFold is an artificial intelligence system known for predicting the structures of well-established model organism proteins based on their amino acid sequences. The structure obtained from AlphaFold for Cav-1 had high confidence for 90% of the amino acids, except near the N-terminus. Furthermore, due to the availability of ITGα3 crystal structures of ITG-3 in RCSB-PDB, the structural coordinates for ITGα3 exhibited very high confidence for more than 95% of the residues, despite the large size of the protein. The quality of the structural coordinates obtained using AlphaFold was further assessed using PDBsum [36, 37]. Cav-1 structural prediction showed that 100% of the residues were in feasible regions in the most favorable or additional allowed regions, and 98% of the ITGα3 protein residues were predicted to be in the most favored or additional allowed regions. Protein-protein interactions between Cav-1 and ITGα3 were predicted using the ZDOCK server (https://zdock.umassmed.edu/) and visualized using PyMOL [38, 39]. The ZDOCK server employs an algorithm to generate hard-docking results, also known as rigid-body search, which identifies potential interaction surfaces between proteins based on the surface charge and structural compatibility. The results with the highest predicted interaction values and feasibility were exported in a suitable image format.

### Coimmunoprecipitation

To validate the physical interactions between Cav-1 and ITGα3, as predicted by in silico observations, a coimmunoprecipitation experiment was conducted using a Pierce Coimmunoprecipitation Kit (Cat# 26149; Thermo Fisher, Waltham, MA). Approximately 3 × 10^6^ 4T1 WT cells were seeded in T-75 flasks and incubated overnight at 37 °C in a 5% CO_2_ incubator. The next day, the medium was replaced, and the cells were allowed to grow for 24 h in an incubator. Cells were then lysed in IP lysis buffer, and insoluble cell debris was removed by centrifugation at 13,000×*g* for 10 min at 4 °C. The clear supernatant was carefully collected, and protein estimation was performed using the Pierce BCA Protein Assay Kit (Thermo Fisher, Waltham, MA), following the manufacturer’s protocol, with some modifications [40]. For immunoprecipitation, 500 μg of protein per sample in a 200 μL volume was used. Pre-cleaning was performed using a control agarose resin slurry. An equal volume of precleared protein lysate was transferred to a fresh microcentrifuge tube, and 2 μg of Caveolin-1 antibody (Cat# 3238, Cell Signaling Technology) and normal rabbit IgG (Cat# 2729, Cell Signaling Technology) were added, followed by slow end-to-end mixing on a rotator at 4 °C overnight. The Amino Link Plus coupling resin was equilibrated at room temperature and washed three times in IP lysis buffer. The lysate (with antibody) was transferred to the Amino Link with coupling beads and incubated for slow end-to-end mixing on a rotator at 4 °C for 4 h. After incubation, the beads were separated by centrifugation and washed thrice. Immunoblotting samples were prepared by adding 1x loading dye to the beads and heating them at 95 °C for 6–8 min. The samples were probed with mouse anti-Cav-1 (Cat# sc-53564; Santa Cruz Biotechnology) and mouse anti-ITGα3 (Cat# sc-374242; Santa Cruz Biotechnology) antibodies.

### Cav1/ITGα3 gene knockdown

A gene-silencing experiment was conducted in 4T1 cells to confirm the role of Cav1/ITGα3 in epithelial cell migration. Cav-1 (Cat# sc-29942), integrin α3/ITGA3/CD49c (sc-63320), and scrambled control siRNA (sc-37007) were obtained from Santa Cruz Biotechnology Inc. (Dallas, Texas, USA). The TransIT-X2® Dynamic Delivery System (Cat# MIR 6004) (Mirus Bio LLC) was used for siRNA transfection following the manufacturer’s protocol. Briefly, 0.15 × 10^6^ 4T1 cells were seeded in a 6-well plate and incubated overnight at 37 °C in a CO_2_ incubator. After 16–24 h, the siRNA and TransIT-X2® Dynamic Delivery System complex was prepared in Opti-MEM™ reduced serum medium and incubated for 15–30 min. Following complex formation, siRNA complex was added to the cells and incubated for 48 h. After this period, 4T1 cells were synchronized in FBS-free media for 24 h. After synchronization, a wound was scratched in each well using a 200 µL pipette tip. The complete medium was replaced with a fresh medium, and images were captured at a magnification of 4x at 0, 24, and 48 h. These findings were confirmed in the MDA-MB-231 human cell line using human siRNA for Cav1 (Cat#sc-29241) and integrin α3/ITGA3/CD49c (Cat#sc-63319) (Santa Cruz Biotechnology, Inc). Control siRNA-A (sc-37007) was used as control. The wound healing area was determined using ImageJ software. After 48 h, the cells were lysed, and immunoblotting was performed to confirm cell protein and gene silencing.

## Results

### Generation and characterization of Cav-1 knockout 4T1

**Fig. 1:**
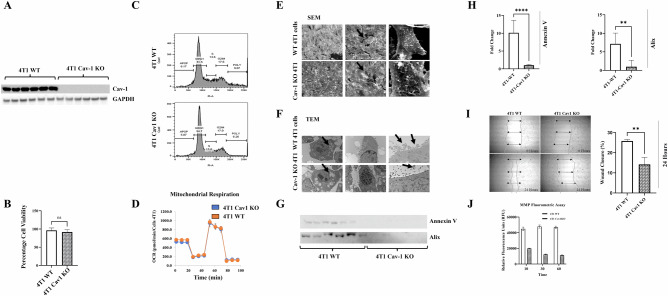
Characterization of the effects of Cav-1 deficiency in 4T1 cells. CRISPR/Cas9 was used to target Cav-1 in 4T1 cells. **A** Immunoblot showing absence of Cav-1 protein expression in purified Cav-1 knockout cells as compared to control 4T1 cells. **B** MTT assay showing that knocking out Cav-1 did not affect cell viability at 48 h. **C** Cell cycle assay showing no significant differences in the diverse phases of cell growth using flow cytometry. **D** The cell mitochondrial stress assay showed no significant effect on mitochondrial respiration in Cav-1 KO mice compared to control 4T1 cells using an Agilent Seahorse XF Analyzer. **E** SEM Image showing comparative cell surface morphology. **F** TEM images show altered membrane morphology and vesicle release. **G** Immunoblot showing reduced expression of exosome markers in the extracellular vesicles isolated from Cav-1 KO cell growth media and control after 24 h. **H** Densitometry data showing reduced expression of Annexin V and Alix in Cav-1 KO conditioned media compared to WT 4T1 conditioned media. **I** Wound healing assay showing reduced migration ability of Cav-1 KO cells compared to control 4T1 cells. **J** MMP fluorometric assay showed significantly reduced secretion of MMPs in Cav-1 KO mice compared to control 4T1 cells in conditioned media. Statistical analysis was performed using unpaired Student’s *t*-test *P < 0.05; ***P < 0.001.

4T1 is a triple-negative murine mammary BC cell line that emulates the characteristics of advanced stage IV cancer in humans. We used CRISPR/Cas9 to generate Cav-1 knockout 4T1 cells, and several colonies were purified and confirmed by immunoblotting (Fig. 1A). Cav-1 protein expression was undetectable by immunoblotting, which was further confirmed by the insertion of GFP into Cav-1 KO cells. Furthermore, Cav-1 KO did not have significant effects on cell growth (Fig. 1B), the cell cycle (Fig. 1C), or mitochondrial stress compared to WT 4T1 cells (Fig. 1D). However, SEM showed that the membrane phenotype of Cav-1 KO cells differed from that of WT 4T1 cells (Fig. 1E), which may be associated with their tumorigenic and metastatic potential [41]. Furthermore, TEM revealed fewer vacuoles in Cav-1 KO cells than in the control (Fig. 1F). We also observed more extracellular vesicles protruding from WT cells than from Cav-1 KO 4T1 cells. This observation was further confirmed by immunoblotting for the exosome markers Annexin V and Alix (Fig. 1G), and densitometric quantification was performed (Fig. 1H). In vitro scratch cell migration assays demonstrated significantly higher migration and wound closure in WT 4T1 cells at 24 h, which was not observed in Cav-1 KO 4T1 cells (Fig. 1I). Furthermore, epithelial cell migration and matrix metalloproteinase (MMP) activation are vital for metastasis [42, 43]. The MMP fluorometric assay revealed higher secretion of MMP in WT cells than in Cav-1 KO-conditioned medium (Fig. 1J). These characteristics of Cav-1 KO cells suggest that Cav-1 plays a role in promoting metastasis.

### Cav-1 knockout affects lung metastasis

**Fig. 2:**
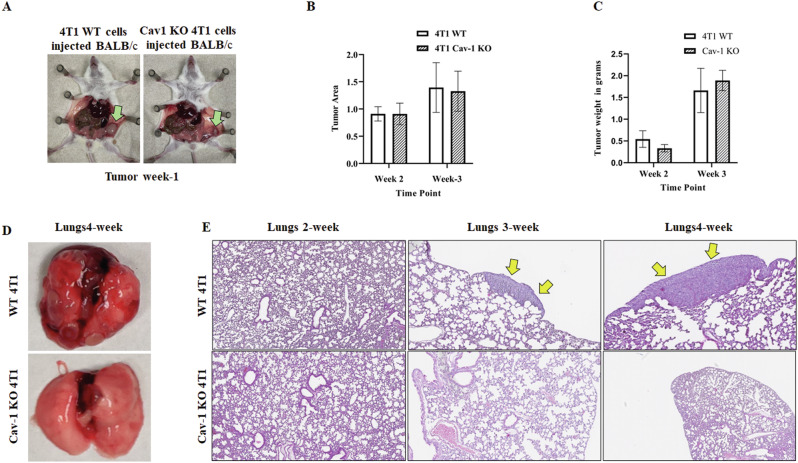
Cav-1 regulates 4T1 growth and lung metastasis in vivo. 4T1 cells were injected into the mammary pads of BALB/c mice. Tumor progression and lung metastasis were followed for 4 weeks: **A** Green arrow in the image showing delayed tumor growth in Cav-1 KO injected mice compared to WT 4T1 injected mice at the end of week 1; **B** comparative tumor area (length × width) at the end of weeks 2 and 3; **C** Graph showing relative tumor weight at the end of weeks 2 and 3; **D** Image showing visible metastatic lesions in the WT lungs at the end of week 4, which were not present in the Cav-1 KO cell-injected mice; **E** H&E staining of lungs, yellow arrows show metastatic foci in WT, but no metastasis was seen in Cav-1 KO cells injected lungs at different time points. Statistical analysis was performed using unpaired Student’s *t*-test *P < 0.05; ***P < 0.001.

Equal numbers of WT and Cav-1 KO 4T1 cells were injected into the mammary fat pads of female BALB/c mice, and tumor growth was observed over time. Five animals per group were used to study metastases from the primary tumor site. One week after injection (week 1), small tumors were detected in mice injected with WT 4T1 (controls), which were even smaller in mice injected with Cav-1 KO cells (Fig. 2A). To understand the molecular mechanisms and kinetics of tumor growth, the animals were sacrificed at the end of weeks 2, 3, and 4. Unlike our observations from week 1, the size of the tumors harvested from both control and Cav-1 KO 4T1 cell-injected mice did not show significant differences at the end of weeks 2 and 3. The tumor areas and masses in both groups were comparable (Fig. 2B, C). At the end of week 4, clear metastatic tumor lesions were visible in the lungs of the WT control group but not in the Cav-1 KO group (Fig. 2D). Additionally, Dr. M. E. White, a pathologist, evaluated the H&E-stained lungs of the animals to detect lung metastases. At the end of week 2, multiple metastatic foci (0–7 per lobe) were observed in the lungs of the control animals. Several of these metastatic foci were 50–108 μm in size, with the widest ranging from 57 to 150 μm. Indistinct lesions, including lymphocytic inflammation, hemorrhage, and edema, were also identified in the lungs of WT and Cav-1 KO mice. At the end of week 3, more prominent metastatic foci (1–5 per lobe) were detected, ranging from 225 μm to 728 μm in controls which was further supported by significantly high number of 4T1 cells in the lungs of WT as compared to Cav-1 KO, 4T1 injected mice (Supplementary Fig. 3). At the end of week 4, the number of metastatic foci was 2–10 per lobe, ranging from 1.12 mm to 2.79 mm in controls. However, no metastatic foci were observed in Cav-1 KO 4T1 injected mice at the end of weeks 2, 3, and 4 (Fig. 2E). Taken together, our data suggest that the absence of Cav-1 expression limits the metastatic potential of tumors from the primary mammary site to the lungs.

### Cav-1 deficiency affected the expression of genes involved in epithelial cell migration

**Fig. 3:**
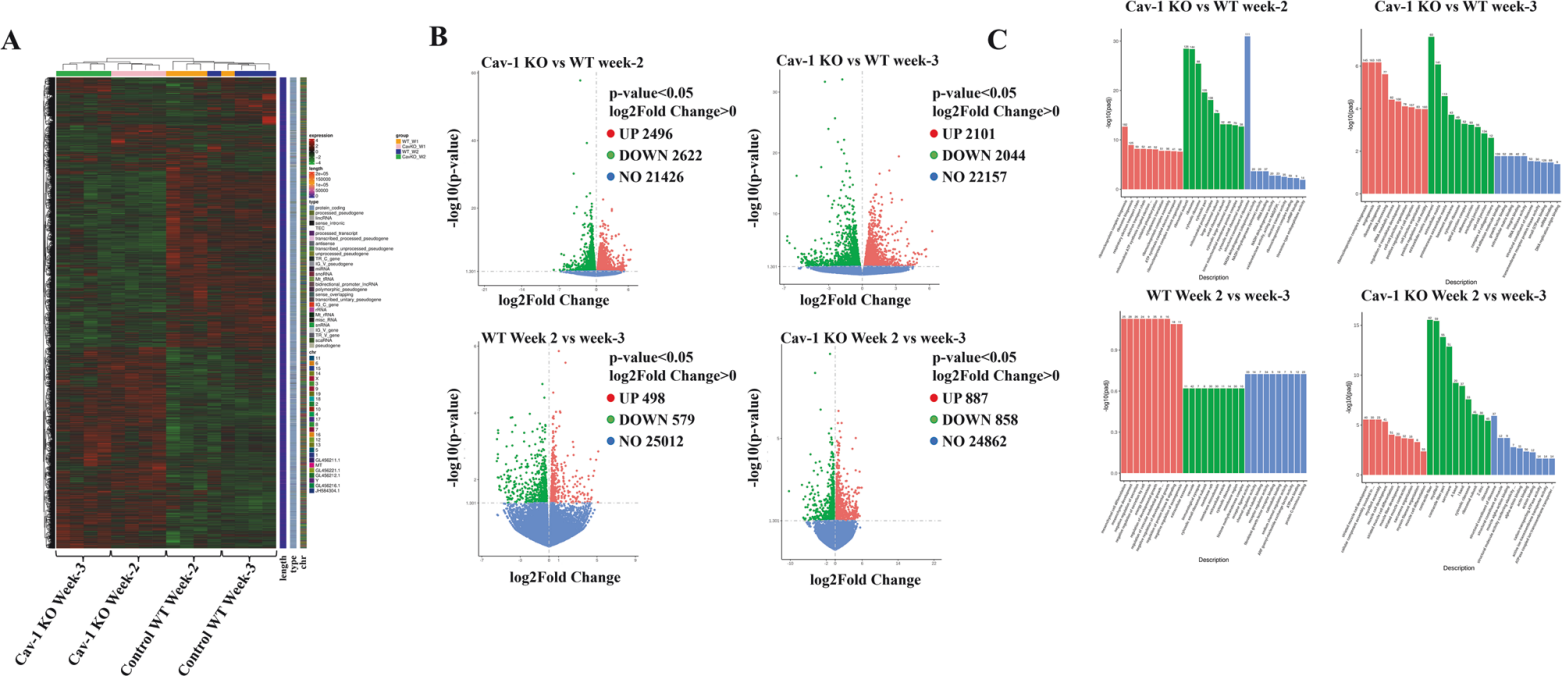
RNA was isolated from each tumor group, and mRNA sequencing analysis was performed. **A** Heat map showing cluster analysis of differentially expressed genes in Cav-1 KO and WT 4T1 cell-induced tumors; **B** Volcano plot showing differential gene expression between Cav-1 KO and WT 4T1 cell-induced tumors at weeks 2 and 3. DEG were also plotted between WT with WT and KO with KO between week 2 and 3 **(C)**. DEG (padj < 0.05) were categorized into biological processes (BP), cellular component (CC), and molecular function (MF). DESeq at threshold DESeq2, *P*-value ≤ 0.05.

IHC data revealed reduced lung metastasis in mice injected with Cav-1 KO cells compared to WT controls. To better understand the molecular mechanisms and gene expression patterns, we performed mRNA sequencing of primary tumors obtained from WT control and Cav-1 KO 4T1 injected mice. Gene expression levels were estimated using quality sequence counts assigned to the genome. FPKM (expected number of fragments per kilogram of transcript sequence per million base pairs sequenced) considers both sequencing depth and gene length when counting fragments, making it the most widely used method for gene expression analysis. Following gene expression analysis, the Pearson correlation coefficient between samples was found to be very close to 1, confirming the reliability of the data (Supplementary Fig. 1A). Principal component analysis (PCA) of FPKM obtained from the Cav-1 KO and WT 4T1 groups demonstrated differences between groups and duplication of intragroup samples, indicating that the Cav-1 KO and WT groups were separated; however, samples from the same groups clustered together (Supplementary Fig. 1B). Once gene expression was quantified, statistical analysis was performed using DESeq at a threshold DESeq2 *P*-value of ≤ 0.05. The number of DEGs among all groups and pairs is shown in a Venn diagram (Supplementary Fig. 2A, B). Statistical data analysis revealed 5118 DEG between Cav-1 KO and WT mice at the end of week 2, 2496 of which were upregulated and 2622 were downregulated. Similarly, at the end of week 3, 4145 genes were differentially expressed, with 2101 up-regulated and 2044 down-regulated. We also identified DEGs within the same group (WT vs. WT and Cav1 KO vs. Cav1 KO) at different time points (Fig. 3B). Cluster analysis was performed using hierarchical main grouping of different gene sets, in which similar expression patterns could be grouped together. Cluster analysis showed that the DEGs in Cav-1 KO and WT 4T1 cells could be grouped into two clusters (Fig. 3A). Enrichment analysis was conducted to determine the biological significance of DEGs. ClusterProfiler [44] software was used for enrichment analysis. GO term enrichment showed that DEGs (padj < 0.05) were involved in the major categories of cellular components, molecular functions, and biological processes (Fig. 3C).

**Fig. 4:**
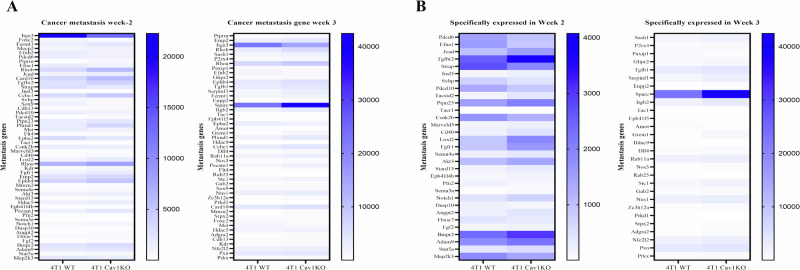
Knocking out Cav-1 affects gene expression involved in epithelial cell migration. **A** Heatmap demonstrating that knocking out Cav-1 expression significantly affected 53 genes at week 2 and 49 genes at week 3. **B** Heatmap showing DEG, which is known to be involved in epithelial cell migration and expressed at specific time points in either week 2 or week 3. DESeq at threshold DESeq2, *P*-value ≤ 0.05.

To understand the functions, biological pathways, and disease information associated with the DEG, KEGG enrichment analysis was performed using padj < 0.05, and 20 KEGG pathways were selected for display. The primary focus of this study was to understand the role of Cav-1 in lung metastasis by identifying 91 genes at week 2 and 89 genes at week 3 that were significantly differentially expressed between Cav-1 KO vs. WT 4T1 injected tumors known to be involved in epithelial cell migration. Of these, 53 of 91 genes at week 2 and 49 of 89 genes at week 3 were significantly different after *P*-value adjustment (Fig. 4A).

**Fig. 5:**
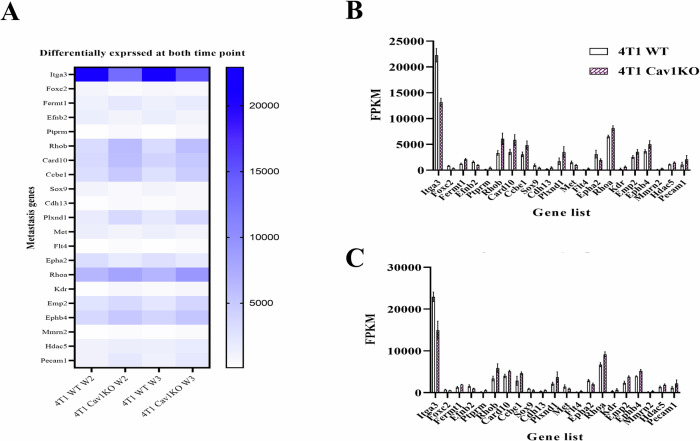
The deletion of Cav-1 significantly altered the expression of epithelial cell migration genes at weeks 2 and 3. **A** Heatmap showing a set of epithelial migration genes that might play a regulatory role in Cav-1 KO-hampered lung metastasis at weeks 2 and 3. The *Y*-axis represents the metastatic genes, whereas the *X*-axis represents the different groups. **B** Graphical representation of epithelial migration genes at week 2 and **C** week 3. *Y*-axis represents FPKM value, and *X*-axis represents the gene name. DESeq at threshold DESeq2, *P*-value ≤ 0.05.

To elucidate the molecular mechanisms underlying the observed lung metastasis from the primary tumor in WT mice as compared to Cav-1 KO at weeks 2 and 3, as revealed by our IHC data, we conducted differential gene expression analysis in the primary tumor. We found 31 genes in week 2 and 28 in week 3 that were significantly differentially expressed at individual time points (Fig. 4B), while 21 genes were differentially expressed at both time points at week 2 and week 3 (Fig. 5A), which are graphically represented in Fig. 5B (Week-2) and 5C (Week-3).

**Fig. 6:**
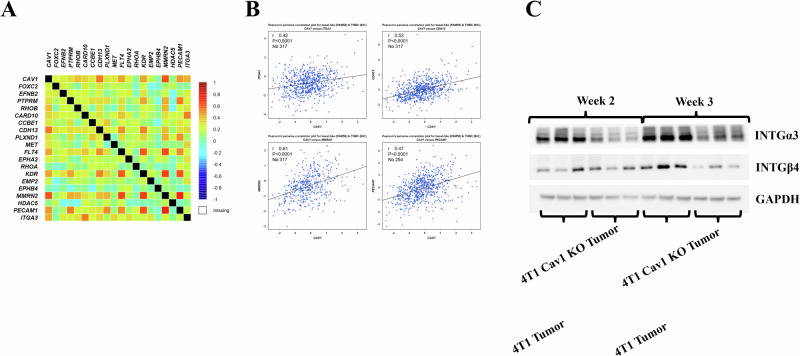
Data mining from the Human Breast Cancer Gene Expression Miner (Breast Cancer Gene-Expression Miner v4.8) database showed that Cav-1 expression was correlated with patient outcome and mortality in TNBC. **A** Correlation analysis between Cav-1 and 21 significantly differentially expressed genes in the human breast cancer patient database. **B** Significant correlation between Cav-1 and CDH13, KDR, MMRN2 (*n* = 317), and PECAM-1 (*n* = 254) expression in TNBC patients with breast cancer. **C** Immunoblot showing variable gene expression between Cav-1 KO and WT 4T1 injected tumors, which was more prominent at the end of week 3.

Correlation data analysis of 4421 human TNBC patients (BC gene expression miner database) revealed a significant correlation between Cav-1 and many of the 21 commonly expressed genes, including ITGα3, CDH13, MMRN2, KDR, and PECAM1 (Fig. 6A, B). The reduced expression of ITGα3 in Cav-1 KO 4T1-induced tumors and its localization to the plasma membrane led us to speculate that ITGα3 plays an essential role in Cav-1-mediated lung metastasis. To support the mRNA data, we performed immunoblotting of ITGα3, which showed significantly reduced expression in Cav-1 KO tumors compared to WT (Fig. 6C). Further computational in silico modeling and coimmunoprecipitation were performed to understand the possible interactions between Cav-1 and ITGα3.

**Fig. 7:**
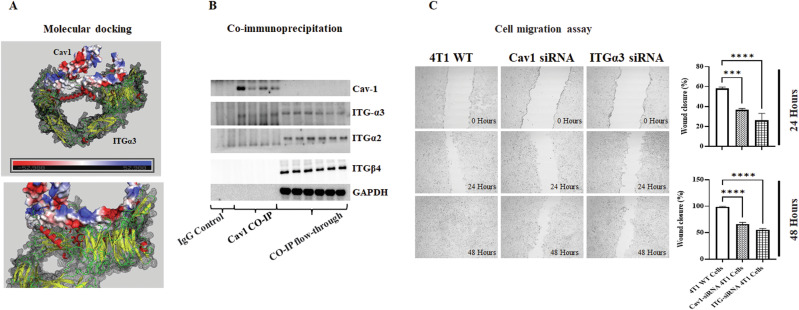
Cav-1 promotes epithelial cell migration by interacting with ITGα3. **A** Interaction between Cav-1 and ITGα3, as predicted using the in silico protein interaction *ZDOCK server (*https://zdock.umassmed.edu/*)*. **B** Coimmunoprecipitation of Cav-1 and ITGα3 confirmed their physical interactions. **C** Wound healing assay of Cav-1 and ITGα3 knock down 4T1 cells showed impaired epithelial cell migration after 24 and 48 h of scratch formation. Graph showing wound closure in the different groups at these time points. Statistical analysis was performed using unpaired Student’s *t*-test *P < 0.05; ***P < 0.001.

In silico modeling and coimmunoprecipitation analysis revealed a possible interaction between Cav-1 and ITGα3, which are both membrane proteins that play crucial roles in cancer progression. AlphaFold was used to predict the structures of Cav-1 and ITGα3. The ZDOCK server was used to examine their interaction with AutoDock VINA in the background (Fig. 7A). We discovered an interaction between Cav-1 and ITGα3 via a hydrophobic patch on Cav-1. Coimmunoprecipitation experiments using an anti-Cav-1 antibody supported the coupling results, showing ITGα3 co-precipitation with Cav-1, demonstrating the physical interaction between Cav-1 and ITGα3 (Fig. 7B). Furthermore, the roles of ITGα3 and Cav-1 in cell migration and invasion were verified using a cell migration assay (wound healing assay) after knocking down ITGα3 and Cav-1 expression using siRNA.

### Knocking down Cav-1 and ITGα3 inhibits the migration of 4T1 and MDA-MB-231 cells

Knockdown of Cav-1 and ITGα3 expression hampers migration of 4T1 cells. The cell migration assay is a standard method used to study the in vitro migration and invasion capabilities [45]. Cav-1 silencing inhibited cell migration, as evidenced by significantly fewer wound closures than in WT 4T1 cells 24 h after wound creation (Fig. 7C). Interestingly, after 48 h, the wound gap was completely sealed in the culture plate containing WT 4T1 cells, but scratches were still visible in Cav-1 silenced 4T1 cells. However, compared to Cav-1 KO 4T1 cells, Cav-1 silenced cells exhibited wound healing capability (Fig. 7C), potentially due to a smaller reduction in Cav-1 expression resulting from its silencing (knockdown) rather than a knockout, which eliminated Cav-1 expression. Similarly, ITGα3 knockdown resulted in reduced migratory potential compared to that of WT 4T1 cells (Fig. 7C). These findings were further confirmed in human MDA-MB-231 Cells (Supplementary Fig. 4). Like Cav-1 knockdown, ITGα3 knockdown also resulted in reduced migratory potential compared to WT 4T1 cells, demonstrating the potential role of Cav-1 interaction with ITG-a3 in lung metastasis.

## Discussion

The role of Cav-1 in the metastasis to the lungs remains unclear, despite links between Cav-1 dysregulation, poor prognosis, and increased metastatic potential in BC. This study elucidated several key aspects of the BC metastatic cascade, focusing on Cav-1-mediated BC metastasis to the lungs. Cav-1 KO 4T1 cells and a syngeneic mouse model of advanced BC were used to study Cav-1 function in BC progression and metastasis in a physiologically relevant system. The effects of Cav-1 deficiency on cellular behaviors, including migration, wound closure, MMP secretion, and gene expression, were also examined.

Our results provide the first in vivo evidence that Cav-1 KO reduces the migratory abilities of BC cells. Importantly, Cav-1 deficiency did not significantly affect primary tumor growth in vivo but markedly decreased lung metastasis in mice bearing Cav-1 KO 4T1 cells compared to WT 4T1 cells. Lung pathology analysis showed the progression of lung metastasis over time in WT mice, with metastatic foci present at weeks 2 and 3, whereas no foci were detected in mice with Cav-1 KO cells which was further confirmed by growing single-cell suspension of the lungs in 6GT supplemented complete growth media at the end of week-3. Visible lung lesions were observed at week 4 in WT mice but not in KO mice. Transmission electron microscopy revealed a significant reduction in vesicle budding in Cav-1 KO 4T1 murine BC cells compared to WT controls. Since cav-1 is an essential structural component of caveolae plasma membrane invaginations involved in endocytosis and transcytosis [46, 47]. These results suggest that Cav-1 regulates vesicular transport in BC cells, and through modulation of vesicle formation, it enhances the metastatic potential of BC cells.

Previous studies have implicated Cav-1 and lipid rafts in cancer metastasis by promoting cellular behaviors involved in metastatic cascades. Yamaguchi et al. showed that decreasing Cav-1 expression or disrupting lipid rafts attenuated MMP expression and invadopodia formation in cultured cancer cells [48]. Furthermore, Gupta et al. demonstrated that targeted disruption of lipid rafts/Cav-1 or the mevalonic acid pathway reduced the invasiveness and metastatic potential of pancreatic cancer cells [49]. Cav-1 overexpression has also been associated with increased MMP production and invasiveness in hepatocellular and ovarian carcinoma models [50, 51]. Our current findings provide further validation of the functional importance of Cav-1 and lipid rafts in cancer metastasis. Specifically, we revealed that genetic ablation of Cav-1 expression in murine 4T1 BC cells induced changes in cell membrane phenotypes and reduced extracellular vesicle secretion. Critically, Cav-1 knockdown also suppressed cell migration via wound closure, in vitro. Overall, the results of our study and those of previous studies established that Cav-1 and lipid rafts enable metastatic cascades.

It is important to highlight the contrasting findings between our study, which shows that Cav-1 knockout reduces lung metastasis in breast cancer models, and the findings of Sloan et al. [28], which links Cav-1 much lower expression with increased metastasis, highlighting Cav-1’s context-dependent role in cancer. Differences in experimental approaches, cell lines, and specific contexts may contribute to the contradictory results regarding the role of Cav-1 in breast cancer metastasis.

To further understand the molecular mechanisms underlying Cav-1’s premetastatic effects, we performed transcriptomic profiling of tumors from Cav-1 KO and wild-type mice. It is well established that heritable morphological traits associated with genomic and transcriptomic phenotypes correlate with distinct tumorigenic and metastatic potential in vivo [41]. Our transcriptomic analysis of BC tumors in Cav-1 KO and WT 4T1 cell-conditioned mice revealed significant alterations in the expression of genes involved in epithelial cell migration, suggesting a possible mechanism by which Cav-1 modulates the metastatic potential. Our analysis also revealed several DEGs between Cav-1 KO and WT 4T1 tumors. Overall, 5118 and 4145 genes were differentially expressed between Cav-1 KO and WT mice at the end of weeks 2 and 3, respectively. Using GO term enrichment analysis, we identified a subset of 21 genes that consistently showed differential expression in lung metastasis at two different time points. Correlation analysis revealed a significant association between Cav-1 and DEG, including CDH13, FLT4, KDR, MMRN2, PECAM1, and ITGα3.

Our study also investigated the correlation between Cav-1 expression and patient outcomes, including mortality, in TNBC patients. By employing data mining analysis of the human BC gene expression miner database, we observed a significant association between Cav-1 expression and genes involved in cell migration, including CDH13, KDR, MMRN2, and PECAM-1 [52, 53]. This finding substantiates the role of Cav-1 in regulating BC cell migration. A notable observation from our mRNA data was the elevated expression of VEGF-C/Flt-4 axis genes, such as VEGFR-2 (KDR) and PECAM-1, in Cav-1 knockout (KO) tumors. Intriguingly, lung metastasis was reduced in Cav-1 KO tumors, suggesting that Cav-1 may govern lung metastasis through alternative signaling pathways. Notably, previous studies have associated CDH13 and MMRN2 with cell invasion and proliferation inhibition [54–56].

Another critical finding of our study is the interaction between Cav-1 and ITGα3, a membrane protein that plays a pivotal role in cell adhesion and migration. ITGα3 has emerged as a promising candidate for further investigation because of its significant correlation with Cav-1 expression in human TNBC samples and its localization to the plasma membrane. In silico modeling and coimmunoprecipitation experiments confirmed this interaction, and subsequent knockdown of ITGα3 expression resulted in impaired migration and invasion of 4T1 cells, similar to the effects observed in Cav-1 KO cells. Therefore, our results suggest that the interaction between Cav-1 and ITGα3 is a key factor in promoting BC metastasis. Supporting the role of ITGα3 in epithelial migration and tumor growth, Kurozumi et al. demonstrated that ITGα3 targets tumor suppressor microRNAs such as miR-223, miR-124-3p and the miR-199 family [57]. Restoring miR-223 expression or knocking down ITGα3 and ITGβ1 significantly prevented cancer cell migration and invasion. Similarly, Idichi et al. showed that ectopic expression of miR-124-3p or siRNA-mediated knockdown of ITGα3 and ITGβ1 reduced the migration and invasion of PDAC cells [58]. Moreover, ITGα3 expression is regulated by the miR-199 family in HNSCC cells, and the knockdown of ITGα3 inhibits the migration and invasion of HNSCC cells. Notably, overexpression of ITGα3 has been predicted to be associated with poor patient survival [59].

Our study has several implications for understanding the molecular mechanisms underlying BC metastasis and developing novel therapeutic strategies. First, identifying Cav-1 as a critical player in promoting metastasis highlights the potential of targeting Cav-1 to prevent or reduce metastasis in BC patients [60, 61]. Notably, several small-molecule inhibitors and monoclonal antibodies targeting Cav-1 have been developed and are currently under investigation in preclinical and clinical studies [62, 63]. Second, the discovery of a possible interaction between Cav-1 and ITGα3 suggests that simultaneously targeting both proteins could represent a more effective strategy for inhibiting metastasis. Integrins have long been recognized to be essential for cell adhesion, migration, and invasion. ITGα3, in particular, has been implicated in promoting metastasis in various types of cancers, including BC [32, 64]. Given the interaction observed between Cav-1 and ITGα3 in our study, it is possible that a combination of Cav-1- and ITGα3-targeted therapies could exert a synergistic effect on inhibiting metastasis. However, further studies are needed to investigate this possibility and optimize therapeutic strategies targeting Cav-1 and ITGα3.

Although our study provides valuable insights, several limitations remain that reveal avenues for additional research. Elucidating the precise molecular mechanisms governing Cav-1 and ITG-α3 interactions represents a key challenge. Techniques such as coimmunoprecipitation and proximity ligation assays can help identify post-translational modifications or protein-binding partners that regulate complex formation [65]. Additionally, Cav1 binding assays with ITG-α3 mutants would delineate key residues involved. Mapping downstream signaling pathways using phosphoproteomics and genetic approaches is also warranted, given the known effects of Cav-1 on MAPK, PI3K/AKT, Rho GTPases, and transcriptional networks. Although we have confirmed migration assay invitro in human MDA-MB231 cells, a further detailed study is warranted in different human cell lines to understand the intricate cellular mechanism of Cav-1 and ITG-α3 in lung metastasis in humans. Expanding into more clinically relevant models, such as patient-derived xenografts or organoids that capture inter-tumor and intra-tumor heterogeneity, will better approximate the complexity of human BC. In addition, studies on the potency, selectivity, and pharmacokinetic properties of Cav-1 and ITGα3 inhibitors will enable their clinical translation.

## Conclusions

This study revealed the role of Cav-1 in BC metastasis, particularly in lung metastasis, without affecting primary tumor growth. Sequencing data from the primary tumor shows that knocking out Cav-1 affects the expression of several epithelial migration genes, which might contribute to lung metastasis. Reduced expression in the primary tumor and interaction between Cav-1 and ITGα3 suggest a mechanism for metastatic enhancement. These findings offer new insights into the molecular dynamics of breast cancer metastasis and underscore the potential of targeting Cav-1 and ITGα3 in therapeutic strategies.

### Limitations and future direction

In this study, we used a 4T1/BALB/c syngeneic TNBC mouse model, focusing on the association between Cav-1 and ITGα3. Most of our findings are from 4T1 cells in the BALB/c syngeneic breast cancer model. Our strategy involves utilizing the 4T1 TNBC mouse cell line to induce tumor development in mice rather than relying on a xenograft model. However, ongoing investigations in our laboratory include the use of different TNBC human and mice cell lines, including Cav-1 and ITGα3 knockout 4T1 cells, MDA-MB-231, and other xenograft models to elucidate the intricate mechanisms underlying the Cav-1/ITGα3 axis in the context of lung metastasis.

## Supplementary information

Supplementary Figure legends

Supplementary_Figure_1

Supplementary_Figure_2

Supplementary_Figure_3

Supplementary_Figure_4

## Data Availability

The data are available from the corresponding author upon reasonable request.
