# Supplementary material for: Caveolin-1 knockout mitigates breast cancer metastasis to the lungs via integrin α3 dysregulation in 4T1-induced syngeneic breast cancer model
Source: Cancer Gene Ther. 2024 Sep 7;31(11):1658–68. doi: 10.1038/s41417-024-00821-4 (PMC11567888; doi:10.1038/s41417-024-00821-4)
Supplement: Supplementary file 1 — Supplementary Figure legends [file 41417_2024_821_MOESM1_ESM.docx]

**Supplementary Fig 1:**

**Cav-1 KO and WT 4T1 injected tumor gene expression profiles showed a strong positive correlation between each pair of samples.** A. Heatmap showing Pearson correlation coefficient of all 16 samples from all four groups. The expression levels of the entire gene set from each pair of samples were used to calculate the correlation coefficient. **B.** Principal components analysis showed distinct variation among Cav-1 KO and WT injected tumor group. However, expression variation was not well separated between week2 and week3 in both Cav-1 KO and WT tumors.

**Supplementary Fig 2:**

**Venn diagram showing the number of DEGs between Cav-1 KO and WT 4T1 cell-injected tumors. A**. Diagram represents the number of common and differentially annotated genes expressed among different groups in Cav-1 KO, and WT at end of week 2 and 3. **B** Venn diagram showing comparative number of DEGs in Cav KO and WT groups at week 2 and 3.

**Supplementary Fig 3:**

**Data showing the number of 4T1 cells grown in 6-GT supplemented complete media.** The graph shows a significantly higher number of cells grown in 4T1 WT lungs than from Cav-1 KO-injected mice. Statistical analysis was performed using unpaired Student’s t-test * P < 0.05; *** P < 0.001.

**Supplementary Fig 4:**

***Data from MDA-MB 231 confirms the finding from 4T1 cells:*** **(A)** Wound healing assay of Cav-1 and ITGα3 knockdown in human MDA-MB 231 cells confirmed the impaired epithelial cell migration after 24 of scratch formation. **(B)** Immunoblotting and densitometry showing the knockdown efficiency. *Statistical analysis was performed using unpaired Student’s t-test * P < 0.05; *** P < 0.001.*
