# Supplementary figures and images for: Caveolin-1 knockout mitigates breast cancer metastasis to the lungs via integrin α3 dysregulation in 4T1-induced syngeneic breast cancer model
Source: Cancer Gene Ther. 2024 Sep 7;31(11):1658–68. doi: 10.1038/s41417-024-00821-4 (PMC11567888; doi:10.1038/s41417-024-00821-4)

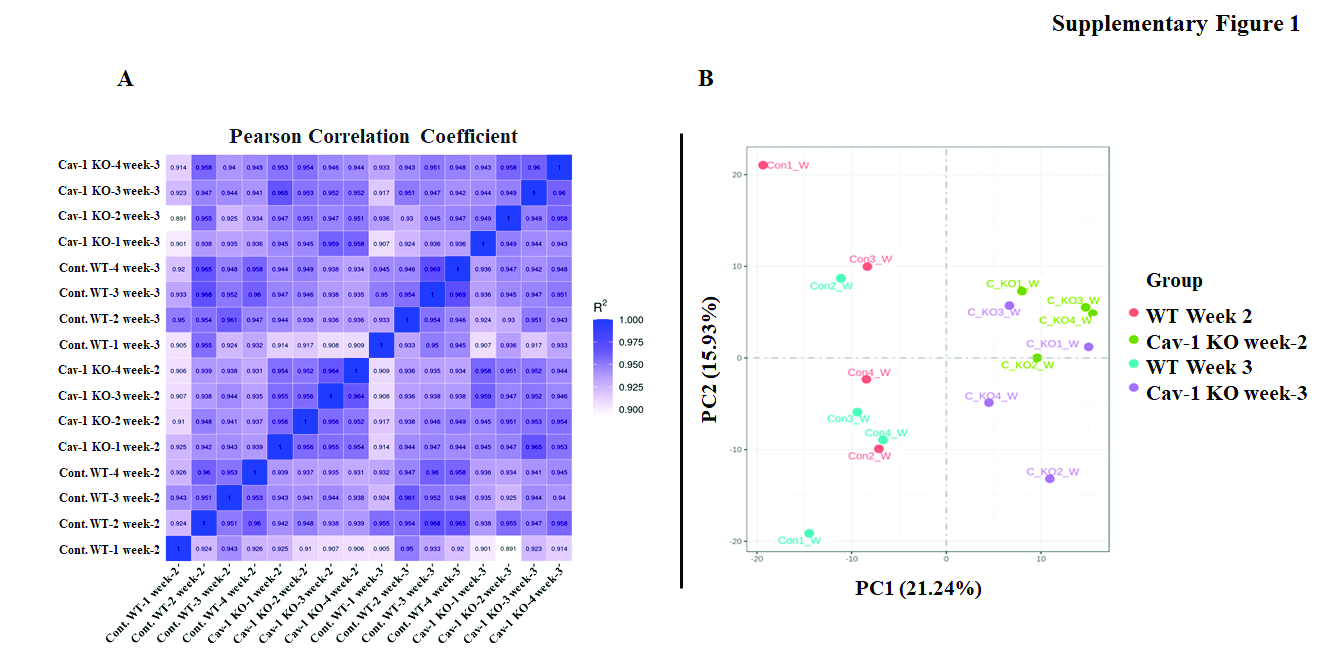

Supplement: Supplementary file 2 — Supplementary_Figure_1 [file 41417_2024_821_MOESM2_ESM.tif]

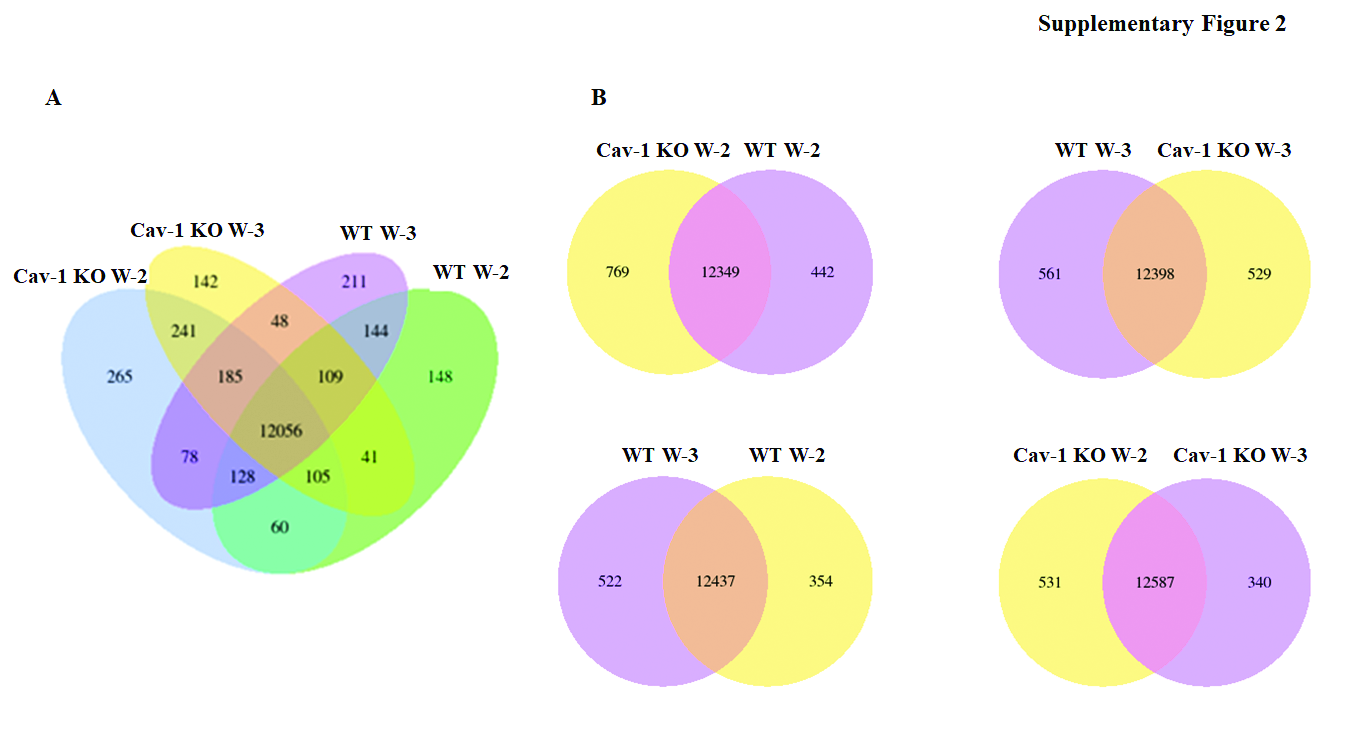

Supplement: Supplementary file 3 — Supplementary_Figure_2 [file 41417_2024_821_MOESM3_ESM.tif]

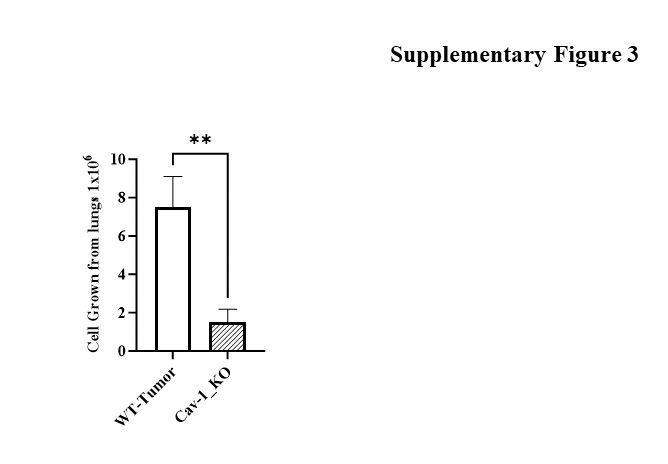

Supplement: Supplementary file 4 — Supplementary_Figure_3 [file 41417_2024_821_MOESM4_ESM.jpg]

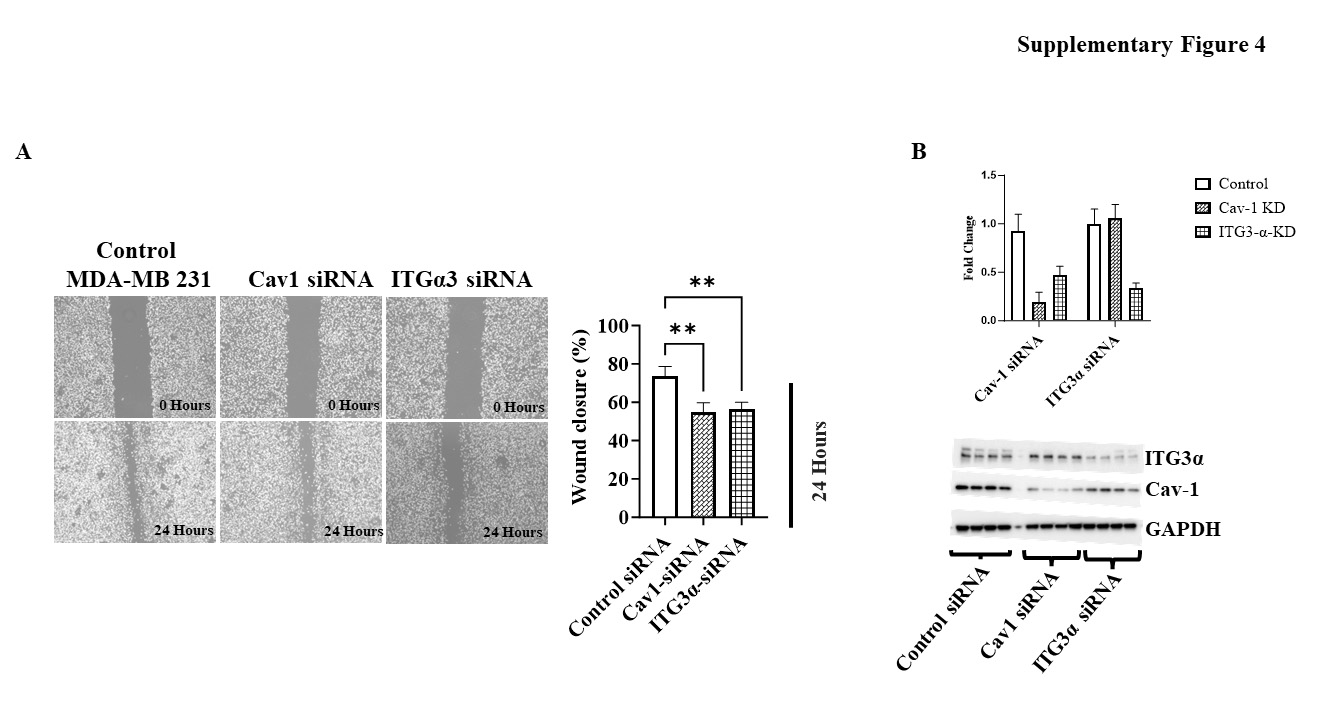

Supplement: Supplementary file 5 — Supplementary_Figure_4 [file 41417_2024_821_MOESM5_ESM.jpg]
